# A Psychosocial Critique of the Consequences of the COVID-19 Pandemic on UK Care Home Staff Attitudes to the Flu Vaccination: A Qualitative Longitudinal Study

**DOI:** 10.3390/vaccines12121437

**Published:** 2024-12-20

**Authors:** Adaku Anyiam-Osigwe, Thando Katangwe-Chigamba, Sion Scott, Carys Seeley, Amrish Patel, Erika J. Sims, Richard Holland, Veronica Bion, Allan B. Clark, Alys Wyn Griffiths, Liz Jones, Adam P. Wagner, David J. Wright, Linda Birt

**Affiliations:** 1 Norwich Clinical Trials Unit, University of East Anglia, Norwich NR4 7TJ, UK; t.katangwe@uea.ac.uk (T.K.-C.); carys.seeley@nsft.nhs.uk (C.S.); e.sims@uea.ac.uk (E.J.S.); v.bion@uea.ac.uk (V.B.); adam.wagner@uea.ac.uk (A.P.W.); 2 School of Healthcare, University of Leicester, Leicester LE1 7RH, UK; s.scott@leicester.ac.uk (S.S.); d.j.wright@leicester.ac.uk (D.J.W.); linda.birt@leicester.ac.uk (L.B.); 3 School of Economics, University of East Anglia, Norwich NR4 7TJ, UK; amrish.patel@uea.ac.uk; 4 Medical School, University of Exeter, Exeter EX1 2LU, UK; richard.holland3@exeter.ac.uk; 5 Norwich Medical School, University of East Anglia, Norwich NR4 7TJ, UK; allan.clark@uea.ac.uk; 6 School of Medicine and Population Health, University of Sheffield, Sheffield S1 4ET, UK; alys.griffiths@sheffield.ac.uk (A.W.G.); lizjones1@live.co.uk (L.J.); 7 NIHR Applied Research Collaboration East of England, Cambridge CB2 8AH, UK

**Keywords:** care homes, COVID-19, influenza vaccination, behavioural theories, process evaluation

## Abstract

**Background/Objectives:** Vaccinating care home staff is essential to protect vulnerable residents by reducing infection risks and creating a safer care environment. However, vaccine hesitancy amongst staff remains a challenge, particularly since the COVID-19 pandemic raised concerns about side effects and vaccination mandates. This study examines how the pandemic influenced flu vaccine hesitancy amongst UK care home staff. **Methods**: Data were collected from the *FluCare* trials conducted over the 2021–22 and 2022–23 winter seasons to explore the impact of concurrent mandatory and non-mandatory COVID-19 vaccination policies on flu vaccine uptake. A total of 52 interviews (21 from the feasibility study and 31 from the randomised control trial) were conducted with care home managers and staff. Thematic analysis identified key themes shaping staff attitudes toward flu vaccination. Results: Four central themes emerged regarding the impact of the pandemic on staff attitudes and the contextual influences shaping vaccine hesitance: (i) tension between autonomy and morals in vaccination decisions; (ii) the COVID ‘craze’ and the displacement of the flu vaccine; (iii) the role of the COVID ‘craze’ in staff vaccine fatigue; and (iv) conspiracies, (mis)information, and the significance of trust. Psychosocial theories on decision making and health behaviour were used to further interpret the findings. **Conclusions:** Our findings suggest that post-COVID-19 interventions in care home setting should address the issues of autonomy, vaccine fatigue, and trust to enhance vaccine uptake. Understanding these factors could support more effective strategies to address hesitancy amongst care home staff in future vaccination campaigns.

## 1. Introduction

There are multiple theories around why people decide not to have vaccinations, termed vaccine hesitancy [1], yet vaccination for healthcare staff is deemed essential to protect themselves from transmissible illness and to ensure they do not infect vulnerable patients (World Health [2]). Vaccinating care home staff is especially important to protect vulnerable older residents. Vaccination reduces the likelihood of infections being transmitted to residents with weaker immune systems and lowers the overall infection rate in care homes, creating a safer environment for both staff and residents [3,4]. However, despite evidence supporting the importance of staff vaccinations, there is still a reported vaccine hesitancy amongst staff [1]. The World Health Organisation recorded that the average flu vaccination rate amongst UK care home staff is below 30%, although they recommend a 75% vaccination rate to reduce cross-infection. Research has demonstrated that hesitance towards the flu vaccination may be attributed to a lack of knowledge about the flu itself; perceived vulnerability to the flu; low social pressure; a general negative attitude to vaccinations; limited access to vaccination facilities; and socio-demographic characteristics [5,6].

Staff behaviours and beliefs around the COVID-19 vaccine resonate with enablers and barriers to those identified in other examples of vaccine hesitancy. Recent studies have investigated the role of the COVID-19 pandemic and the enforcement of the mandatory vaccination policy for UK care home staff (Dec 2021–Mar 2022). Whilst the mandate substantially decreased the proportion of care home workers who remained unvaccinated for COVID-19, staffing levels were reduced [7], and staff reported predominantly negative reactions [8,9,10]. Moreover, Friedrich and Forbes [11] interviewed care home staff in England and reported that the enablers for COVID-19 vaccine uptake included a personal desire to protect others and social influences. The barriers highlighted included fears about side effects, rapid vaccine development that sparked safety concerns, and feeling pressured when the vaccine was mandatory. The data collected from care home staff in England also identified concerns about vaccine safety in relation to pregnancy and the risk of allergic reactions [8,12].

There remains a notable gap in understanding the potential transferability of attitudes about COVID-19 vaccinations towards other vaccines, such as the flu vaccine. Understanding the transferability is especially crucial in settings like care homes, where vaccine uptake is already low. This paper reports findings of a secondary analysis of interview data collected from care home staff within the process evaluation in the FluCare study. This secondary analysis aimed to understand the impact of the pandemic, including COVID-19 vaccination polices, on staff perceptions of flu vaccinations. We now briefly describe the FluCare intervention and timelines of the study to contextualise the methods and results.

### Setting the Study Context

The FluCare study had the primary aim of increasing flu vaccination rates amongst care home staff in England. The multi-faceted intervention was informed by the behavioural change theory. Full details of the intervention are in the randomised control trial (RCT) protocol paper [13]. The intervention consisted of co-developed information posters and videos that aimed to address concerns about the safety and reinforce the importance of flu vaccination for all staff. Materials were provided to care home managers to share with staff. The information was designed to include a representation of people in different care home roles, of different ages, and from different ethnic backgrounds. The second element of the intervention was that free vaccination clinics were offered in the care home to overcome any potential cost barriers. The third element was a financial incentive to care homes that achieved a staff vaccination rate over 70%. To be eligible for the study, care homes had to report ≤40% staff vaccination in the previous flu season.

**Figure 1:**
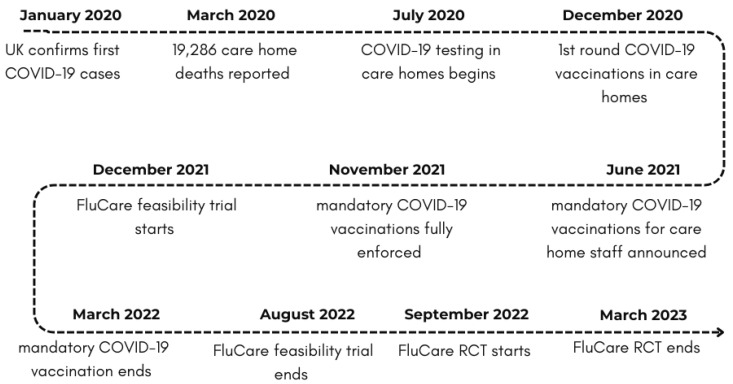
Timeline of feasibility and RCT during the pandemic (Jan 2020–March 2023).

The FluCare feasibility trial was conducted between November 2021 and August 2022, where the media messages and research were centred around COVID-19-related information (e.g. mortality rates, public health guidelines, vaccination deployment and distribution, etc.). The feasibility trial also overlapped with the enforcement of the COVID-19 vaccination mandates in UK care homes (Dec 2021–Mar 2022) [14]. The FluCare randomised control trial (RCT) was conducted between October 2022 and March 2023 (full protocol details blinded for peer review) when care home COVID-19 restrictions were completely lifted and there were no mandatory requirements for COVID-19 vaccination amongst staff, although the public health message remained to take all COVID-19 vaccinations offered. Figure 1 shows a timeline of the events. A process evaluation was embedded in both FluCare studies, thereby providing data on staff perceptions of flu vaccinations at two distinct points as public health narratives changed.

## 2. Methods

### 2.1. Study Design

This study is a secondary qualitative analysis of the data collected within the FluCare process evaluations of the feasibility trial and the RCT (Trial Registration: ISRCTN 22729870, IRAS: #316820). Both process evaluations sought to examine contextual factors that affected the implementation of the intervention. The study design was informed by the Medical Research Council’s framework on process evaluation for complex interventions [15]. FluCare protocols papers are reported elsewhere, including details on the care homes and how the control and intervention groups and sample size were determined [13,16].

### 2.2. Sample and Recruitment

In the feasibility study, staff from all 10 care homes (2 intervention and 8 control) were invited; in the RCT, purposeful sampling was used to identify a subset of 15 care homes (13 intervention and 2 control) representative in characteristics (number of beds and staff and type of registration) to the 37 intervention care homes and 38 control care homes. Participant information sheets were emailed to the care home managers to ask whether they would consider taking part in an interview and to distribute the participant information to all their staff. Care home staff directly approached the research team to express an interest. We intended to purposively sample staff for their age, ethnicity, and job role, but there were limited expressions of interest, so all who expressed an interest were interviewed.

### 2.3. Data Collection

Consent was taken virtually prior to each interview. Ethical approval was received by the University of East Anglia, Faculty of Medicine and Health Ethics Committee (study approval number ETH2122-2419). Semi-structured interviews were conducted via MS Teams by AAO, TKC and CS within four weeks of each study finishing. While the topic guide included questions related to the FluCare intervention and existing vaccination initiatives, this analysis focused on questions about staff and manager awareness and perceptions of flu vaccinations, the ease of receiving flu vaccinations, and the impact of the pandemic on the willingness to get vaccinated. Recordings were transcribed verbatim, and anonymised transcripts were uploaded onto NVivo.

### 2.4. Data Analysis

Following Clarke and Braun’s [17] thematic analysis methodology, the secondary data were analysed by AAO. The researcher focused on how participants spoke about the impact of COVID-19 on flu vaccinations. The data were first considered and coded within two discrete data sets (feasibility and RCT). This enabled us to examine the differences and similarities in staff accounts across the two periods. Then, by combining the data set, sub-themes were developed and arranged into four distinct themes. It was not possible to undertake participant validation due to the time elapsing between data collection and this secondary analysis. The themes were refined through regular discussion with the process evaluation team. The themes were discussed with the patient and public involvement (PPI) group. who predominantly agreed with the lines of argument, with some suggested refinements. After the initial meeting with the PPI group, the final themes and illustrative quotes were reviewed and agreed upon by the process evaluation team. Supplementary Figure S1 contains a collation of the coded data extracts, organised into categories and themes. The illustrative quotes were selected to reflect the consensus within each theme. After the themes were finalised, we explored various theoretical frameworks to explain these findings. This is discussed in the ‘Theoretical Application’ section of this report.

## 3. Results

In the feasibility process evaluation, we undertook interviews with 11 staff and 10 managers, and in the RCT, 13 managers and 18 staff. Data directly referring to views on flu vaccinations were drawn from these interviews [*n* = 49]; three CH staff from the RCT intervention arm were excluded, as they did not discuss anything relating to COVID-19. Table 1 and Table 2 show the feasibility and RCT participant demographics, respectively. The demographics of ethnicity and age was not collected in the feasibility study. Supplementary Table S1 presents more detailed information, including staff roles.

Four themes were developed: (i) tension between autonomy and morals in vaccination decisions; (ii) the COVID ‘craze’ and the displacement of the flu vaccine; (iii) the role of the COVID ‘craze’ in staff vaccine fatigue; and (iv) conspiracies, (mis)information, and the significance of trust. The first theme explores how the COVID-19 vaccination mandates created a conflict between staff moral obligations to protect residents and their desire for autonomy, and its impact on flu vaccination uptake. The second and third themes focus on how the COVID ‘craze’, which refers to the extensive focus on COVID-19 during the pandemic, compared to the flu, (a) lowered the threat severity of the flu and (b) resulted in the perception of overvaccination and vaccine fatigue. The final theme explores how the spread of misinformation about vaccines during the pandemic adversely affected staff perceptions of the flu vaccine and increased the demand for information trustworthiness to encourage vaccinations amongst staff.

### 3.1. Tension Between Autonomy and Morals in Vaccination Decisions

In both the feasibility study and RCT, the staff and managers were unhappy with the mandatory COVID-19 vaccine policy, recalling the pressure they felt from the government to take the vaccine to remain employed.

We were also threatened with, and it happened, that if you weren’t vaccinated by… the 16th November, you had to leave. And three members of staff left, good members of staff. And I was pleading with them to stay and get vaccinated, and they left.

(CHS_002_feasibility)

According to managers, the COVID-19 mandates and the associated sanctions decreased staff willingness to take the flu vaccine.

I do believe that the COVID-19 mandatory vaccinations for care staff had a massive impact on the uptake of the flu vaccine this year… Because that’s what the staff was reporting to me when they weren’t going for it. They weren’t having it because they’d already had enough vaccines.

(CHM_003_main)

They also suggested that because of backlash from the mandates, staff were intentionally refusing the flu vaccine because it was not mandatory.

Personally, I’ve been running this home five years, and I’ve known some staff that had the flu vaccine religiously that haven’t had it this time… Yes. [the COVID-19 mandates] had a negative impact, yes… no one’s bothered since [the flu vaccine] weren’t mandatory, yes.

(CHM_003_main)

Further conversations with the participants on the importance of being vaccinated in their role revealed that most understood that as care home staff, being vaccinated is important for protecting themselves and their residents. For example, CHS_006_feasibility shared that “when you work in this sort of sector… you’ve got vulnerable people with various illnesses and that and we do have a duty of care to them”; similarly, CHS_004_feasibility expressed that although “the last booster made [her] feel quite poorly [she] would have the next booster as well, to protect [the residents] definitely”. Both accounts demonstrate that staff view receiving vaccinations as a moral obligation.

However, despite this desire to protect residents, the enforcement of the COVID-19 mandates discouraged staff from getting the flu vaccine. They perceived mandates as unethical, suggesting that they should be allowed to choose whether they received the vaccines.

Our choice obviously was taken away from us because we work in the care sector and now you don’t have to have [vaccines] to work in the care sector so it’s a bit like, we were all forced to otherwise we would have lost our jobs and now we don’t have to so why should you have something that you don’t have to have?

(CHS_018_main)

In this instance, this staff member suggests that the resistance to vaccinations may be an effort to regain the autonomy lost during the mandatory policy period. This notion is supported by one manager who expressed that opposition towards the flu jab is largely driven by staff enforcing self-governance.

Obviously, at the beginning, when we had to have the [COVID-19] vaccines, even though the NHS didn’t, it’s always, “Why are you picking on care homes?… And now you’re making me have the flu.” It’s like, “No, no, no, we’re not making you have the flu.” … and then we’d all been encouraged to have [the flu jab] but even by the time the third and the fourth [Flu vaccination clinic] was coming now, everyone’s saying, “No, you can’t force me to have it. I’ve had enough. I want to be able to make the choice.”.

(CHM_002_main)

Managers further explained the ongoing psychological implications of forcing staff to take the COVID-19 vaccine. They compared the care home sector to other healthcare sectors, where there was no mandatory policy in effect, which ultimately made them feel undervalued.

I think that then doesn’t come down to necessarily vaccines themselves or COVID-19 vaccines or flu vaccines, but more around staff’s general feeling of being undervalued as a job role, being under valued in pay, being undervalued …it was made mandatory and then it wasn’t made mandatory in health care … I think that there may be a bit of a backlash… I suppose and that’s a way of them kicking back I suppose.

(CHM_001_main)

In these accounts, there appears to be a link between autonomy and staff perception of value, such that the ability to make informed choices influenced whether the staff felt respected and valued in their roles.

It may be that the desire for vaccine autonomy and the perception of being undervalued may have taken precedence over their moral responsibilities, such that although the staff members understood that getting vaccinated was the morally ‘right’ thing to do, they prioritised the ability to make vaccination decisions for themselves.

### 3.2. COVID ‘Craze’ and Displacement of Flu Vaccine

The COVID ‘craze’ refers to the extensive focus on COVID-19 during the pandemic, including heightened public and media attention, public discourse, and healthcare priorities. For participants, this ‘craze’ prompted comparisons between COVID-19 and the flu as an illness and their vaccinations, which mostly resulted in a preference for the COVID-19 vaccine over the flu vaccine.

Throughout the pandemic, the staff explained that discussions in the care home and on the news were predominantly about COVID-19, and nearly all staff recalled the severity of illness caused by COVID-19, whereas, at the time, many could not recall that of flu.

I don’t know anyone who’s been really ill from flu. But I know people get ill from flu, but it’s a normal illness, not anything that’s going to make me think oh I need to protect myself … With COVID-19 there a visual, you see people getting really ill, it’s different isn’t it. It makes you want to take it. But if don’t know anybody who’s had the flu, why would you take it?

(CHS_008_feasibility)

The referral to the flu as a ‘normal’ illness suggests that the exposure to the severity of COVID-19 decreased the participant’s feelings of being susceptible to the flu.

Other responses mirrored this participant’s, whereby the staff felt that COVID-19 was more dangerous than the flu, which resulted in a preference for the COVID-19 vaccine over the flu jab. CHS_017_main explained that “more people were frightened of getting COVID-19 than they were the flu, so they were more likely to get the booster rather than the flu jab”. Arguably, the belief that the COVID-19 jab is stronger than the flu vaccine, arguably, is another consequence of this COVID ‘craze’.

Comparatively, the staff in the feasibility study were more likely to prefer to receive the COVID-19 vaccine to the flu vaccine. The staff believed that the COVID-19 vaccine was stronger than the flu vaccine.

Some of them who have taken the COVID-19 vaccine they are reluctant to take the flu vaccine in some cases … I was trying to tell them that all these years we have been taking, they say “yes, I know, but we have taken the stronger vaccine so unless it didn’t protect us, so next year we will take [the flu vaccine]. That sort of thinking. (CHM_007_feasibility).

And so they felt that taking the flu vaccine after receiving the COVID-19 vaccine was unnecessary.

Now we all have COVID-19 vaccination, so I don’t think flu jab is needed in the future as well. So, what’s the point of flu jab when we have COVID-19 vaccinations, … Yes, before COVID-19 vaccination I agree, we should take [the flu vaccine]. But after COVID-19 vaccination I don’t think it makes any sense to take now flu jabs.

(CHS_007_feasibility)

Arguably, this decreased demand for the flu vaccine is the result of the contextual differences of both time periods. The feasibility study was conducted during the peak of the pandemic, so staff would have received more exposure to the severity of COVID-19 in their care homes and on the media compared to the RCT.

### 3.3. Role of the COVID ‘Craze’ in Staff Vaccine Fatigue

Another consequence of the COVID-19 ‘craze’ was the perception of overvaccination—staff perception that they have been subjected to too many vaccines—which caused some of them to experience vaccine fatigue.

Interestingly, the feeling of vaccine fatigue was more prominent amongst the staff in the RCT, further highlighting the impact of context on these findings. The extended period between the onset of the pandemic and the FluCare RCT likely exposed the staff to numerous vaccine promotions, contributing to increased fatigue.

One manager suggested that there was little engagement with the intervention components of FluCare because the staff were tired of hearing about vaccinations.

I think the positives in the [FluCare] idea was good. The fact that we were trying to arrange clinics in the home was good, to actually bring them to us, rather than us say, “Go to your GP or go to your pharmacy.” But I just think we just fell really short of the mark, and again, that’s the timing. That’s the timing of it, and that’s COVID-19, and that’s due to the fatigue. Nothing to do with the FluCare promotion or study at all.

(CHM_002_main)

As this staff member explains, the staff were tired of the repeated advertising and persuading to get their COVID-19 jabs and boosters, and so they were demotivated from getting the flu vaccine.

I suppose some people may be getting tired of vaccines, you know, there’s been quite a lot recently…–if you took all the COVID-19 ones. I suppose there’s been about five now altogether, if everybody took everything, and a flu on top, maybe people are thinking that’s too much.

(CHS_003_main)

The perception that taking both COVID-19 and flu vaccines was ‘too much’ was also accompanied by the belief that taking both vaccines simultaneously may be unsafe.

Everyone’s a little bit more hesitant, and especially with having both at the same time, because they were offering us [the COVID-19 and flu vaccines] at the same time. And [staff] were like “no it hasn’t really been tested, has it?” I know they say it has but until you have a long-term thing then they’re worried about two different vaccinations at the same time, what are they going to do? Are they going to react?

(CHS_005_feasibility)

As a result, staff were more likely to choose between vaccines, and this decision was largely based on individual factors such as age, underlying illnesses, and the perception of susceptibility. For example, CHS_006_main explained that she only receives the flu vaccine because she is more likely to get the flu than COVID-19.

I made the choice this last time of having the flu and not the COVID-19. I wasn’t going to have them both. I had the flu thinking, well, I’ve had enough COVID-19 vaccines, touch wood I haven’t had COVID-19, so I’m just one of those people that have been very lucky. I’m having the flu one because I’m more likely to get the flu than, I think, the COVID-19.

### 3.4. Conspiracies, (Mis)information, and the Significance of Trust

‘Conspiracies’ surrounding the COVID-19 vaccine negatively influenced staff perceptions of the flu vaccine and willingness to get the flu jab, as staff explained that the information—or misinformation—shared during the pandemic about the COVID-19 vaccine increased their scepticism towards the flu vaccine. One staff member said,

You get all the social media don’t you where they say that they’re going to add stuff into the flu vaccine to cover COVID-19 and all that. Because obviously some of our staff didn’t want the COVID-19… so they’re worried that they’re then going to put something in there which made them a little bit worried.

(CHS_005_feasibility)

The staff members shared some of the information that was released about the COVID-19 vaccinations; for example, one said “I hear loads of people say you really don’t know what’s in it, it could be a microchip, the government” (CHS_004_feasibility), and another “I heard that… in COVID-19 vaccination… there is [a] pig fat” (CHS_007_feasibility). Such negative rumours about the COVID-19 vaccine increased vaccine hesitancy towards the flu vaccine.

The staff also described the impact that misinformation about the AstraZeneca vaccine had during the pandemic:

With the controversy that we all had the AstraZeneca… where they were claiming that it was perfectly safe, there’s no evidence of the blood clots… and they adamantly said that, and they were told that by [the governmental department] Infection Control, the same people that are telling them to have the flu vaccination. So, they were told by those same people that there was no issues, and then it all came out that, actually, there were, and it got taken off the market. By that time, [staff] felt they had been put in danger by people that they trusted, and they do not trust them anymore.

(CHS_005_main)

These accounts suggest that misinformation about vaccinations led to a lack of trust, which, in turn, discouraged the staff from getting the flu vaccine.

Notably, the relationship between misinformation and lack of trust as it relates to vaccination intention was observed more between staff in the feasibility study, which is another indication of a contextual influence on vaccination behaviour choices.

The staff in the feasibility study were more expressive of the difficulty in accessing accurate information during the pandemic and how the false information has decreased their trust in the more reliable sources. One staff member said, “I think the main problem is that there is so much b******t in the world these days the people are not trusting the things that are true” (CHS_003_feasibility).

This emphasis on trust in information dissemination was also evident in staff recommendations for how to increase flu vaccine uptake in care homes. The staff commonly suggested that word of mouth from well-informed sources about the flu vaccine would be most convincing.

People are used to the same poster and the same leaflets, and we’ve seen it in the NHS hospitals and the posters are everywhere … I think that the better thing that you can do is have proponents of it that will talk about [the flu vaccine] in coffee times, over clearing up, looking after someone … And I think that that does more than the same old leaflet or poster … so I think it’s the spoken voice, the reasoning, and the discussion that people will have that will, if anything, perhaps get those last two or three members of staff through to saying yes to [the flu vaccine].

(CHS_002_feasibility)

Arguably, this personalised approach to intervention delivery demonstrates a superseding role of trust in whether accurate information concerning vaccines is enough to persuade staff vaccine intentions.

## 4. Discussion: Theoretical Application

This study examined the impact of the COVID-19 pandemic on flu vaccination uptake and the response to the FluCare intervention amongst care home staff. Capturing care home staff perceptions and reasoning about flu vaccination within a global pandemic provides a novel way of understanding how macro- and micro-contextual factors shape vaccine hesitancy. The four themes highlight some factors that may underpin this impact, which include (i) the desire for autonomy; (ii) overexposure, threat severity, and mental fatigue; and (iii) source trustworthiness. As observed, these factors influence the endorsement of the flu vaccine following the pandemic in unique ways. In this section, these factors will be explored in relation to existing psychosocial theories. The wider implications on flu vaccine-targeted interventions are discussed in the concluding section.

### 4.1. Desire for Autonomy in Decision Making

Conversations with care home staff concerning the impact of the COVID-19 mandates revealed an increased resistance to the flu vaccination. The psychological reactance theory [18] may explain this phenomenon. Reactance is a motivational arousal that occurs when an individual’s behavioural freedoms are reduced or threatened. This arousal is typically accompanied by anger and a negative cognition, which results in cognitive and behavioural efforts to re-establish freedom [19]. When mandates are accompanied by sanctions (e.g., dismissal), both freedom threat perceptions and reactance increase [20].

**Figure 2:**
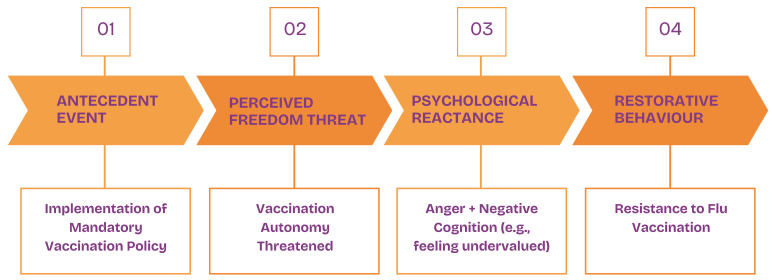
Application of psychological reactance theory.

As depicted in Figure 2, we argue that the enforcing of the COVID-19 vaccine mandates in care homes and attached sanctions led to a high perception of freedom threat to bodily autonomy for staff. This perceived freedom threat triggered a reactance, anger, and a negative cognition. In this instance, the negative cognition is the perception of being undervalued, caused by the mandates being solely enforced in the care sector. The response to this reactance is the observed increased resistance towards the flu vaccine, which may have been the staff’s attempt to re-establish freedom.

Studies support that reactance is a significant predictor of non-compliance to different COVID-19 preventive measures, such as washing hands, social distancing, wearing face masks, and vaccine motivations [21,22,23]. A recent study has also shown that mandate-caused reactance increased the avoidance of both the COVID-19 vaccination and an unrelated chicken pox vaccine, demonstrating that behavioural reactance can extend beyond the reactance eliciting factor, i.e., the COVID-19 mandates affecting attitudes towards other vaccinations [24]. Thus, the theory might explain staff resistance to the flu vaccine following the COVID-19 vaccination mandates.

### 4.2. Overexposure and Risk Perception

Exposure to the severity of COVID-19 in care homes and on the media heightened care home staff risk perception [25]. Our results indicated that the staff had a greater fear of the consequences of COVID-19 infection than flu infection. Consequently, they prioritised the COVID-19 vaccine over the flu vaccine. Such behaviour can be explained by the protection motivation theory (PMT) [26]. The PMT suggests that the likelihood of engaging in a protective behaviour, such as getting vaccinated, depends on beliefs about the efficacy of the protective behaviour itself and the threat posed by the event. Engaging in the protective behaviour is determined by two parallel processes: threat and coping appraisal. Threat appraisal is determined by perceptions of threat severity, susceptibility, and benefits of harmful behaviour. Coping appraisal is determined by beliefs about protective behaviour efficacy, self-efficacy, and barriers to behaviour change. Individuals are most likely to engage in protective behaviour when the threat and coping appraisal is high [27].

**Figure 3:**
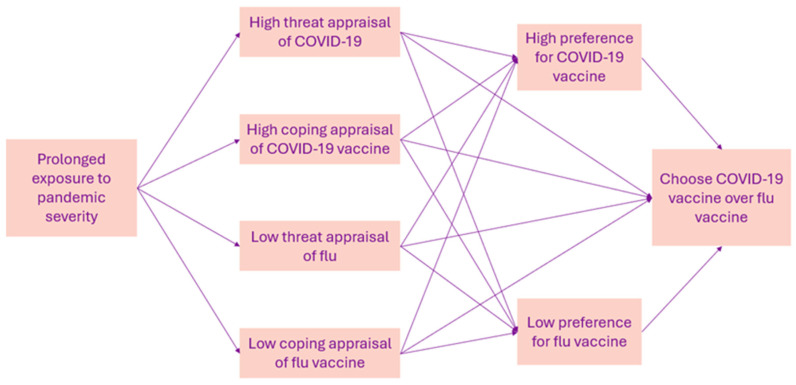
Application of protection motivation theory.

Based on our findings, we suggest that the care home staff experience of the pandemic resulted in simultaneous appraisal processes of both COVID-19 and the flu, such that the staff had a high threat appraisal of COVID-19 and a high coping appraisal of the COVID-19 vaccine alongside a lowered threat and coping appraisal of the flu and its vaccine. Figure 3 illustrates different appraisal patterns that may have led to staff preference for the COVID-19 vaccine over the flu vaccine, as observed in the study. Whilst the PMT has not been used to analyse flu vaccine uptake following the pandemic, studies support that the constructs within the threat and coping appraisal predict vaccination intention of both the flu and COVID-19 vaccination, respectively [28,29].

### 4.3. Fatigue and Risk Perception

Mental fatigue from COVID-19 discussions, prevention messages, and vaccination campaigns increased the resistance towards the flu vaccine. The WHO describes this pandemic fatigue as a gradual demotivation to adhere to the recommended protective behaviours, being influenced by emotions, experiences, and perceptions [30]. Research supports that it can be caused by repeated exposure [31,32] and that it increases vaccine hesitance and hinders the transition from vaccination intent into action [33,34]. In this instance, mental fatigue may have transferred onto the flu vaccination, decreasing staff intention to receive the flu jab. This possibility has been supported by another study that showed that exhaustion due to measures taken against COVID-19 was negatively associated with flu vaccination intention amongst nurses [35].

Motivation is an important factor in explaining the impact of mental fatigue on decision making. Intrinsic motivation stems from internal factors like personal enjoyment, while extrinsic motivation arises from external incentives such as rewards or punishments; both have been directly linked to vaccination intention [36,37]. It could be argued that the staff lost intrinsic motivation to receive the flu jab, especially after the implementation of mandatory policies that made them feel undervalued. Additionally, they may have found the extrinsic motivation provided by FluCare (e.g., care home renumeration) inadequate. As a result, the staff were deterred from engaging with FluCare and receiving a vaccination.

### 4.4. Source Trustworthiness

Trustworthiness of information was a pervading theme in persuading staff to have the flu vaccine. Trust can impact perceived threat severity, information-seeking behaviour, and disposition towards interventions [38,39]. Trust also mediates the relationship between vaccine literacy and vaccine intention. Vaccine literacy is the ability to understand and make informed decisions about vaccines [40]. There is empirical evidence that a high level of trust in the vaccine improves information-seeking behaviour concerning vaccines, and vaccination intentions [41,42].

Based on these studies and the current findings, we can argue that the spread of false information regarding vaccines by different media sources and the Infection Control Department decreased staff trust in vaccines and the wider healthcare system. Our analysis may also explain why the informative materials of the FluCare intervention had a limited effect on staff attitudes toward the flu and minimally improved their understanding of the purpose of the flu vaccines. It can also explain why the staff placed an emphasis on establishing staff trust in the information that was shared to encourage vaccinations.

### 4.5. Study Strengths and Limitations

While the sample appears representative of the care home workforce in terms of gender and age, the self-selecting nature of participation may have resulted in an over-representation of those with strong views on vaccinations, either positive or negative.

### 4.6. Clinical Implications and Conclusions

The findings and explanatory theories provide important implication for future interventions aimed at increasing flu vaccine uptake following the pandemic. For example, interventions like FluCare that include informative materials might benefit from addressing the appraisal constructs—threat and coping appraisal—that the current study identified as mediators to vaccine uptake. That is, interventions should purposively incorporate materials that increase public awareness of the severity of the flu and their susceptibility to it, which can increase threat appraisal. They should also emphasise the effectiveness of the flu vaccination to boost its coping appraisal. Furthermore, they would need to address the misconceptions, such as beliefs that the COVID-19 vaccine is sufficient protection against the flu.

Additionally, emphasis on trustworthiness in the aftermath of the pandemic highlights the need for a bottom–up approach to information dissemination. This approach involves utilising personalised channels, such as discussions led by trusted professionals or community members, to effectively engage staff and enhance their understanding and acceptance of vaccines. Such strategies have the potential to foster greater confidence in the healthcare system and ultimately improve vaccine uptake.

As the study was a secondary analysis, future projects might consider collecting primary data to directly analyse the impact of the pandemic on flu vaccination intention. This would enable the exploration of other potential themes and explanatory theories, particularly studies that explore individual differences in responses to the flu vaccine following the pandemic, as our study highlighted factors such as age, race, and co-morbidities as mediators to the threat appraisal of the flu and the coping appraisal of the flu vaccine. Recommendations for the wider process evaluation and FluCare main trial have been reported elsewhere. Based on our analysis, future interventions to address public health measures in care home settings will need to consider behavioural legacies formed during the COVID-19 pandemic and adequately address them to enact real change.

## Figures and Tables

**Table 1:** Feasibility study participant demographics.

Characteristics		Managers (n = 10)	Staff (n = 11)
Sex	Female	6 (75%)	7 (70%)
	Missing data	2	1

**Table 2:** RCT participant demographics.

Characteristics		Managers (n = 13)	Staff (n = 18)
Sex	Female	13 (100%)	16 (88.9%)
Age range	20–29	0	1
	30–39	0	3
	40–49	5	5
	50–59	3	8
	60–69	2	1
	Missing data	3	0
Ethnicity	White British	8	16
	White other	2	1
	South Asian	0	1
	Missing data	1	0

## Data Availability

The original contributions presented in this study are included within the article/Supplementary Materials. Further inquiries can be directed to the corresponding author.

## Supplementary Materials

The following supporting information can be downloaded at: https://www.mdpi.com/article/10.3390/vaccines12121437/s1. Figure S1: Thematic Analysis of Coded Data Extracts; Table S1: Extended Participant Demographic Information.
