# Supplementary material for: A Psychosocial Critique of the Consequences of the COVID-19 Pandemic on UK Care Home Staff Attitudes to the Flu Vaccination: A Qualitative Longitudinal Study
Source: Vaccines (Basel). 2024 Dec 20;12(12):1437. doi: 10.3390/vaccines12121437 (PMC11728680; doi:10.3390/vaccines12121437)
Supplement: Supplementary file 1 [file vaccines-12-01437-s001.zip › Supplementary Figure S1.pdf]

CODE
BLUE: FEASIBILITY STUDY
GREEN: MAIN TRIAL
M: MANAGER
S: STAFF

Covid Madatory Policy & Its Consequences on Flu- vax & care

manager/staff experience- what happened to staff & their reaction towards policy

being forced to take covid unless you'd lose your job (S)

covid mandatory policy- being forced to take vax (S)

staff angry about being forced to take vaccines (M)

not about jab itself but about being forced; CHS feel undervalued (M)

forced to have covid vax to keep job; negative attitude towards flu (M)

people felt cornered to take vax (mandatory) (S)

mandatory vac frustrated managers (M)

care staff fed up of being told what to do (M)

anti-vaxxers left job because of covid policy (M)

staff disgruntled by mandatory policy change (M)

staff felt pressured to having vax to keep job- CHM dont want to push for flu because of resistance (M)

social care sector unfairly treated during mandatory period (M)

personal experience with covid and how its affected flu vax uptake (M)

lost staff because of mandatory rules (M)

mandatory period was frustrating for staff (M)

staff sick of being told what to do after covid experience (M)

being forced to get the covid vaccine unless they lose their job (unethical)

CHS negative experience during mandatory period (M)

saying NO (to flu & fluCare)

staff are saying no to flu care because couldnt say no to covid (M)

staff feel forced to take covid not going to take flu vax (M)

resistance to flu vax because of covid mandatory policy (S)

exercising choice (M/S)

consequence of being forced to take covid jab- people are exercising their choice and saying no (S)

staff saying no to flu not based on 'logical' reasons, mainly around mandatory policy (exercising choice) (M)

people can have covid test and flu jab at the same time (which is good) (M)

choice taken away during covid, now exercising choice to say no (S)

staff didnt take flu because they didnt want to feel forced (exercising choice) (M)

carers reluctant to flucare because they were 'made' to take covid vax (M)

staff don't want flu because its not mandatory (exercising choice) (M)

people don't want flu vax after being forced to have covid jab (S)

shouldnt force people to get vax, they become more resistant (S)

mandatory policy made peole angry; now exercising choice to say no (M)

Covid Madatory Policy & Its Consequences on Flu- vax & care

developed negative attitude towards flu vaccine

- people are resistant to flu vax because of covid vaccine (protocol related) (S)
- mandatory vax rules increased resistance for those 'on the fence' (S)
- people are resistant to flu vax because of covid vaccine (protocol related) (S)
- forceful nature of covid protocol affected staff uptake of flu jab (M)
- ethical issue with being forced to take vaccine; effect on vax attitude (M)
- experience of being forced to get vaccine formed negative attitude towards vaccine (M/S)
- first reaction to flu related to anxiety from COVID protocols (M)
- covid is 'still fresh' (M)
- forcing staff not good for flu vax uptake (M)
- covid negatively impacted flu by forcing staff to have vax(why) (M)
- people become more resistant to being forced to take vax- (S)
- the forceful nature of covid hardened staff towards flu (M)
- feeling forced negatively changed staff attitudes towards vaccinations (M)
- staff don't pay attention to flu because of mandatory vax law (M)

'pro mandatory'

- making the flu mandatory (like covid) would make her take flu vax (positive) (S)
- making vaccine compulsory only way people will take flucare (S)
- people will take flu jab is made compulsory like covid (S)
- understanding that mandatory policy was to protect residents (but not in support) (S)
- make flu mandatory like covid because people always have excuses (S)
- making flu mandatory would have increased uptake (M)

wider implications

- people dont want to work in care sector cause of covid (S)
- lack of trust caused my covid means interventions like flu care need to do more than educating (M)
- people have lost faith in the vaccine programmes becuse of covid (S)
- more diffiult to convince people to have the flu because its not mandatory (M)
- lack of trust with policy makers (M)
- mandatory policy made people more resistant to being convinced about vaccinations (S)
- mandatory vax has affected vax in general not just flu (M)

Being a carer and taking vaccinations

- understanding that both flu and covid are for protection (S)
- understanding that vaccine minimises risk (S)
- understanding that vax is for protection (S)
- people had covid vax out of compassion for residents (S)
- understanding of taking vaccine in relation to role as a carer (S)
- Covid increased awareness to protect residents (S)
- covid increased sense of responsibility, so people are more inclined to get the flu jab (S)
- flucare made staff feel important again after covid died down (M)
- complied to rules in the CH (not resistant) (S)
- highlight information related to role as a carer to get people to take vax (S)
- its not about flu or covid but passion for the job (S)
- vaccination important for protecting yourself and resident (M)
- getting vaccine for the sake of residents (role as a carer) (S)
- need to protect residents overrode side effect of vaccine (S)
- has an understanding that vaxs protect others (S)

The impact of Covid on Flu (Covid overshadowing/overpowering flu)

covid vax vs flu vax

- people neglect flu jab because of covid jabs (M)
- people felt that covid vax covered flu (didnt want to have both) (M)
- perception that covid is the stronger vaccine (M)
- staff think covid and flu vaccine do the same thing (M)
- staff believe that they dont need flu jab after covid jab (M)
- covid vax is enough to prevent flu (S)
- staff dont want both because they believe covid is like flu (M)
- perception that covid jab and flu jab are similar (M)
- people recieve covid vax well because its newer than flu (M)
- belief that covid injection replaces flu vaccine (S)
- covid vax made flu vax reduntant (S)
- perception that covid vax and flu vax do the same thing (in relation to lowering your vulnerability) (S)

transference of negative effects of covid vax on flu vax

- side effects from covid discouraged them from getting flu vax (M)
- staff experienced negtive side effects of covid jab so they dont want flu jab (M)
- negative experience with covid made CHS want flu vax (positive) (M)
- increased skepticism about symptoms associated with flu as a result of covid vax symptoms (M)
- increased questioning about flu jab because of covid (M)
- staff more skeptical about contents of flu vax because of covid (M)

Impact on Flucare

- flucare would have been good but covid is dominating (M)
- flucare would have been pushed more if not for covid (M)
- flu clinic is good because theres a lot of emphasis around covid (S)
- covid negatively affecting attitude towards flucare (M)

more discussions/awarenes about Covid

- staff talk about covid more than flu & flucare (S)
- staff no longer cautious about flu season- covid has stolen focus (M)
- spotlight on covid has overshadowed importance of flu vaccine (S)
- side effects of covid vax better explained than flu (M)
- progress with flu development overshadowed by covid (M)
- people are discussing covid more than flu (S)
- COVID overshadowing the impact of flu (i.e mortality rate; severeness of symptoms) (M)
- more flu uptake if covid wasnt around (M)
- messaging about usefulness of jab more effective than flu messaging (M)
- manager less concerned about flu compared to covid (M)
- information at GP is all about covid (S)
- imporance of flu vax not as advertised as covid (M)
- flu needs more advertising because of spotlight on covid (S)
- dangers of covid more apparent than flu (S)
- current emphasis on covid more than flu (M)
- covid more discussed than flu (S)
- covid more advertised than flu (S)
- covid has overshadowed discussions about the flu (M/S)
- CHM pushed for flu vax before covid came (M)

comparisons of covid and flu

- people more scared of covid than flu so they got covid jab (S)
- they had to make covid mandatory because it's more contagious than flu (S)
- flu not as dangerous as covid (S)
- staff more concerned with covid (M)
- covid is same as flu, so no need for flu jab after covid jab (S)
- 'takes chances' with flu more than covid (M)
- covid vax is enough to prevent flu (S)
- staff believe that they dont need flu jab after covid jab (M)
- staff perceive that flu has disappeared because of covid (M)
- people attribute flu symptoms to covid (M)
- covid and flu symptoms are similar (S)
- flu more mild than covid (S)
- covid is new so its more scary (M)
- getting flu jab put on the back burner because covid seen more dangerous (S)
- covid vax made flu vax reduntant (S)

the pandemic 'overshadowing' the severity(?) of flu

- staff talk about covid more than flu & flucare (S)
- spotlight on covid has overshadowed importance of flu vaccine (S)
- people are discussing covid more than flu (S)
- perception that covid is the stronger vaccine (M)
- people more scared of covid than flu so they got covid jab (S)
- staff more concerned with covid (M)
- COVID overshadowing the impact of flu (i.e mortality rate; severeness of symptoms) (M)
- more flu uptake if covid wasnt around (M)
- people felt that covid vax covered flu (didnt want to have both) (M)
- manager less concerned about flu compared to covid (M)
- 'takes chances' with flu more than covid (M)
- staff believe that they dont need flu jab after covid jab (M)
- covid more discussed than flu (S)
- flu needs more advertising because of spotlight on covid (S)
- belief that covid injection replaces flu vaccine (S)
- flucare would have been good but covid is dominating (M)
- people attribute flu symptoms to covid (M)
- flu more mild than covid (S)
- flucare would have been pushed more if not for covid (M)
- getting flu jab put on the back burner because covid seen more dangerous (S)
- flu not as dangerous as covid (S)
- covid vax is enough to prevent flu (S)

choosing between Covid and Flu Vax- staff paranoid about taking both covid and flu at same time

choosing between COVID and Flu vaccine (S)

consequence of taking both vaxx at the same time (M)

couldn't get flu and covid jab on the same day (S)

covid jab came before flu vax; can't have both at same time (M)

having both vax made p feel poorly (M)

'its not good to take both vax together' (M)

not enough evidence that its safe to have covid and flu at the same time (S)

people blame flu jab if they have both jabs at same time (M)

people feel like they have to choose between covid and flu (M)

taking flu and covid jab at same time, staff blamed flu (S)

choice may be based on which disease person is more likely to get (S)

staff are tired of vaccinations

covid experience overwhelming for CHS (M)

covid-fatigue (S)

staff fed up of being vaccinated (M)

covid experiece has made staff tired of vaccinations (M)

staff didnt want to hear message about flucare because of covid-fatigue (M)

decreased demand for flu vax (M)

people learn to live with covid; learn to live with flu (M)

staff have has enough of flu following covid rules&outcome (M)

even staff who normally get flu vax dont want it (vaccine fatigue) (M)

staff put off flu vax cause of the amount of covid vax (M)

staff tired of getting vaccinations (M)

flu care was too soon after covid period to be effective (M)

people tired of vaccines after covid so flucare uptake negatively affected- not because flucare project was bad (M)

multiple covid vaccinations so people may not want flu jab because its too much (S)

staff are jab jaded (M)

Feeling of over-vaccination (vaccination fatigue) because of different COVID vaccines (S)

'too much' vaccinations for the body (S)

vaccine after vaccine- people are vaxxed out (M)

'jab fatigue' hindering conversations about flu (M)

covid jab and flu jab at the same time viewed as 'too much' particularly for very vulnerable people (S)

a lot of covid vax- people dont take flu (M)

people experience vaccination fatifue from COVID (S)

staff feel forced to have the multiple covid jabs (overwhelming) (M)

changes related to covid fatigue not policies (M)

staff have negative attitude towards flu care because of multiple covid vaccines (M)

Conspiracies, (Mis)Information & the Importance of Information

Conspiracies

- microchip in covid jab (S)
- people had the belief that covid vax was scanning people (S)
- covid vaccine can affect pregnancy (S)
- heard that there's pig fat in covid vax (S)
- black people would have more side effects to covid vaccine (S)

Information

- getting people on board with vaccines not simply a matter of access to correct information (S)
- easy to access info that agrees with your view whether its factual or not (S)
- people have strong resistance to covid based on beliefs (S)
- participant is well informed from personal experience about vaccines (S)
- people dont have correct info for accurate risk assessment (S)
- people are more gullible (S)
- CH did not take steps to educate staff about importance of covid vax (S)
- over-information in the world so it's hard to sift out factual ones (S)
- accessibility of the internet makes it easy for people to spread false information and even easier for people to believe it (S)

The Impact of information

- rumors about covid vax made people resistant (S)
- skepticism about covid jab content made people skeptical about whats in the flu jab (S)
- people are more skeptical about what's in flu vaccine after covid controversy (S)
- people skeptical about what's in the flu vax (S)
- people didnt want covid vax because of lack of information (S)
- info on social media about covid vax influencing people's view on flu (S)
- resistance to covid (and by association all vax) due to misinformation and ignorance (S)
- negative news about covid affects flu (S)
- people more worried about what is in vaccinations since covid (M)
- people dont trust 'believe in' scientific evidence (S)
- people anxious about whats in flu vax because of covid (S)
- people resistant to covid because they cannot differentiate between fact and fiction (S)

Implications for FluCare

- FluCare should take more personalised approach to campaigning for flu vax (S)
- information of how jab protects residents and minimal side effects for staff would convince people to get flu jab (S)
- needs to be more information about flu to get people convinced (S)
- evidence that personalised approach works to debunk personal confusion (S)
- people won over with 'correct' information (S)
- personalised approach to flucare intervention (S)
- people are scared to get flu jab because of COVID jab controversy (side effects; newness)- misunderstanding (S)
- how misinformation about covid vax impacts what info flu care should focus on (S)
- FluCare would need a trustworthy source (related to info distrust caused by covid) (S)
- FluCare would work if materials informed staff (S)
