# Supplementary material for: A Psychosocial Critique of the Consequences of the COVID-19 Pandemic on UK Care Home Staff Attitudes to the Flu Vaccination: A Qualitative Longitudinal Study
Source: Vaccines (Basel). 2024 Dec 20;12(12):1437. doi: 10.3390/vaccines12121437 (PMC11728680; doi:10.3390/vaccines12121437)
Supplement: Supplementary file 1 [file vaccines-12-01437-s001.zip › Supplementary Tables S1.docx]

**Table S1.** Extended Participant Demographic Information.

| **Feasibility Study Participant Demographics** | | | | | |
| --- | --- | --- | --- | --- | --- |
| **Manager demographics** | | | | | |
| **Participant ID** | | **Gender** | |  | |
| CHM_001_ feasibility | | Female | |  | |
| CHM_002_ feasibility | | n/a | |  | |
| CHM_003_ feasibility | | Male | |  | |
| CHM_004_ feasibility | | Male | |  | |
| CHM_005_ feasibility | | n/a | |  | |
| CHM_006_ feasibility | | Female | |  | |
| CHM_007_ feasibility | | Female | |  | |
| CHM_008_ feasibility | | Female | |  | |
| CHM_009_ feasibility | | Female | |  | |
| CHM_010_ feasibility | | Female | |  | |
| **Staff demographics** | | | | | |
| **Participant ID** | |  | |  | |
| CHS_001­­_feasibility | | **Gender** | | **Role** | |
| CHS_002_ feasibility | | Female | | Care Assistant | |
| CHS_003_ feasibility | | Female | | Nurse | |
| CHS_004_ feasibility | | n/a | | Maintenance Assistant | |
| CHS_005_ feasibility | | Female | | General nurse | |
| CHS_006_ feasibility | | Female | | Business Administrator | |
| CHS_007_ feasibility | | Female | | n/a | |
| CHS_008_ feasibility | | Male | | Carer | |
| CHS_009_ feasibility | | Male | | Admin | |
| CHS_010_ feasibility | | Female | | Team leader | |
| CHS_011_ feasibility | | Female | | Nurse | |
| **Manager Demographics** | | | | | |
| **Participant ID** | **Study Arm** | | **Gender** | |  |
| CHM_001­­_main | Intervention | | Female | |  |
| CHM_002_main | Intervention | | Female | |  |
| CHM_003_main | Intervention | | Female | |  |
| CHM_004_main | Intervention | | Female | |  |
| CHM_005_main | Intervention | | Female | |  |
| CHM_006_main | Intervention | | Female | |  |
| CHM_007_main | Intervention | | Female | |  |
| CHM_008_main | Control | | Female | |  |
| CHM_009_main | Control | | Female | |  |
| CHM_010_main | Intervention | | Female | |  |
| CHM_011_main | Intervention | | Female | |  |
| CHM_012_main | Intervention | | Female | |  |
| CHM_013_main | Intervention | | Female | |  |
| **Staff Demographics** | | | | | |
| **Participant ID** | **Study Arm** | | **Gender** | | **Role** |
| CHS_001_main | Intervention | | Female | | Senior Carer |
| CHS_002_main | Intervention | | Female | | Activities Co-ordinator |
| CHS_003_main | Intervention | | Female | | Carer (part time)/Cook |
| CHS_004_main | Intervention | | Female | | Head of Care |
| CHS_005_main | Intervention | | Female | | Housekeeper |
| CHS_006_main | Intervention | | Female | | Admin |
| CHS_007_main | Intervention | | Female | | Carer/Cook |
| CHS_008_main | Intervention | | Female | | Senior Care Assistant |
| CHS_009_main | Intervention | | Female | | Advanced Senior Care Assistant |
| CHS_010_main | Control | | Female | | Care Assistant |
| CHS_011_main | Intervention | | Female | | Carer |
| CHS_012_main | Intervention | | Female | | Carer |
| CHS_013_main | Control | | Female | | Housekeeper |
| CHS_014_main | Intervention | | Male | | Senior Care Assistant |
| CHS_015_main | Intervention | | Female | | Deputy Care Manager |
| CHS_016_main | Intervention | | Male | | Night Care Assistant |
| CHS_017_main | Intervention | | Female | | Care Assistant |
| CHS_018_main | Control | | Female | | Deputy Care manager |
